# Predictive Score for Advanced Colorectal Neoplasia Based on Cardiovascular and Colorectal Cancer Risk Factors

**DOI:** 10.3390/jcm13102887

**Published:** 2024-05-14

**Authors:** Lara M. Ruiz-Belmonte, Patricia Carrera-Lasfuentes, Alberto Cebollada-Solanas, Carmelo Scarpignato, Angel Lanas, Carla J. Gargallo-Puyuelo

**Affiliations:** 1 Department of Gastroenterology, Miguel Servet University Hospital, Paseo Isabel La Católica, 1–3, 50009 Zaragoza, Spain; 2 Faculty of Health Sciences, Universidad San Jorge, Villanueva de Gállego, 50830 Zaragoza, Spain; pcarreralasfuentes@gmail.com; 3 Unidad de Biocomputación, Instituto Aragonés de Ciencias de la Salud (IACS/IIS Aragón), Centro de Investigación Biomédica de Aragón (CIBA), 50009 Zaragoza, Spain; acebolladaso.iacs@aragon.es; 4 Department of Health Sciences, United Campus of Malta, MSD 2080 Msida, Malta; carmelo.scarpignato@gmail.com; 5 Department of Gastroenterology, Lozano Blesa University Clinical Hospital, Av: San Juan Bosco, 15, 50009 Zaragoza, Spain; alanas@unizar.es (A.L.); carlajerusalen@hotmail.com (C.J.G.-P.); 6 Institute of Health Research Aragon (IIS Aragon), 50009 Zaragoza, Spain; 7 Centro de Investigación Biomédica en Red de Enfermedades Hepáticas y Digestivas (CIBEREHD), Instituto de Salud Carlos III (ISCIII), 28029 Madrid, Spain; 8 School of Medicine, University of Zaragoza, 50009 Zaragoza, Spain

**Keywords:** colorectal neoplasia, cardiovascular risk, risk factors, predictive score

## Abstract

**Background and Aims**: Cardiovascular disease and colorectal cancer (CRC) are significant health problems and share some risk factors. The aim of our study was to develop and validate a predictive score for advanced colorectal neoplasia (CRN) based on risk factors for cardiovascular disease and CRC. **Materials and Methods:** A cross-sectional study comprising a derivation cohort and an external validation cohort of 1049 and 308 patients, respectively. A prediction score for advanced CRN (CRNAS: Colorectal Neoplasia Advanced Score) was developed from a logistic regression model, comprising sex, age, first-degree family history for CRC, systolic and diastolic blood pressure, total cholesterol, HDL cholesterol, body mass index, diabetes, smoking, and antihypertensive treatment. Other cardiovascular risk scores (Framingham–Wilson, REGICOR, SCORE, and FRESCO) were also used to predict the risk of advanced CRN. The discriminatory capacity of each score was evaluated using the area under the curve (AUC). **Results:** CRN were found in 379 subjects from the derivation cohort (36%), including 228 patients (22%) with an advanced CRN. Male sex, age, diabetes, and smoking were identified as independent risk factors for advanced CRN. The newly created score (CRNAS) showed an AUC of 0.68 (95% CI: 0.64–0.73) for advanced CRN, which was better than cardiovascular risk scores (*p* < 0.001). In the validation cohort, the AUC of CRNAS for advanced CRN was 0.67 (95% CI: 0.57–0.76). **Conclusions:** The newly validated CRNAS has a better discriminatory capacity to predict advanced CRN than cardiovascular scores. It may be useful for selecting candidates for screening colonoscopy, especially in those with cardiovascular risk factors.

## 1. Introduction

Both colorectal neoplasia (CRN) (including adenomas and colorectal cancer [CRC]) and cardiovascular disease (CVD) are prevalent pathologies, resulting in high mortality rates [1]. The leading cause of death worldwide is ischemic heart disease, accounting for 16% of total global deaths [1]. Since the year 2000, the largest increase in deaths is attributed to CVD, which has risen from over 2 million deaths in 2000 to 8.9 million in 2019. If we focus on CRC, it is estimated that by the year 2030, its global burden worldwide will increase by 60% (over 2.2 million new cases and around 1.1 million deaths) [2,3]. Several risk factors have been shown to be related to the development of CRN, which are also risk factors for CVD. The most well-established to date include age [4], male sex [5], smoking [6], type 2 diabetes mellitus, and metabolic syndrome [7].

Because of the high prevalence of both CRC and CVD and the existence of similar risk factors, several studies have suggested that patients with established CVD have a higher risk of developing CRN [8,9]. Based on these studies, it would be reasonable to consider CRC screening in patients with established coronary artery disease but this is not performed in routine clinical practice, probably due to the increased 10-year mortality [10] and the potential increased risk of procedure-related complications. However, CRC screening might be justified in patients who have a higher risk of developing CVD but have not yet developed it. On the other hand, it is expected that approaches to improve modifiable CRC risk factors will have an additive positive impact on CVD prevention, and vice versa.

Within the current framework of personalized medicine, the establishment of predictive risk models for CRN should allow individualized screening strategies. In the last decade, several studies have proposed the use of cardiovascular risk (CVR) scores as tools to predict the risk of CRN [11,12,13], however, many of them were conducted in Asian populations. 

Nowadays, in most countries, CRC screening is carried out in selected patients based on a patient’s age and family or personal history of CRC [14] without taking into account other risk factors. Nevertheless, several other predictive scores for advanced CRN based on genetic variants, environmental/lifestyle factors, or cardiovascular risk factors (CVRF) have also been published [15,16] but their discriminatory capacity was moderate. Therefore, there is a need to improve current CRC screening options.

In trying to address this issue, the main objective of our study was to create and validate a predictive score for advanced CRN (including advanced adenoma and CRC) based on CVRF and other factors previously described as risk factors for advanced CRN.

## 2. Materials and Methods

### 2.1. Study Design and Population

This study comprised two cross-sectional cohorts: a derivation cohort (used to create the new score) which consisted of 1049 Caucasian patients who underwent a colonoscopy (Olympus CF-H190, Olympus Europa, Barcelona, Spain) at the tertiary-level Lozano Blesa Clinical Hospital in Zaragoza, between May 2010 and December 2014, and an external validation cohort of 308 Caucasian patients. Patients from the validation cohort underwent a complete colonoscopy (Olympus CF-H190, Olympus Europa, Barcelona, Spain) between July 2019 and March 2020 at Son Espases University Hospital (a tertiary-level hospital in Palma de Mallorca, Spain). Patients were included at the time of colonoscopy. Inclusion criteria: patients aged ≥18 years who underwent a colonoscopy due to (1) CRC screening in average-risk individuals older than 50 years, (2) digestive symptoms, or (3) non-hereditary familial CRC history. Exclusion criteria included: patient refusal to participate in the study, personal history of CRC or prior polypectomy, family or personal history of hereditary CRC (polyposis or non-polyposis), personal history of inflammatory bowel disease, incomplete colonoscopy (cecal intubation not achieved), poor preparation (Boston scale < 6 points), and patients lacking essential demographic or clinical information for the study. 

### 2.2. Ethical Considerations

All subjects provided informed consent for the study, which was approved by the ethics committee of each participating hospital. This study was conducted in accordance with the ethical standards established in the 1964 Declaration of Helsinki and its subsequent amendments.

### 2.3. Study Variables

Demographic, clinical, and biochemical data were recorded as shown in Table 1 (data from the six months prior to the colonoscopy). Laboratory parameters and weight measurements were obtained after an overnight fast of at least 8 h. Blood pressure measurements were obtained on the day of colonoscopy or, if available, from primary care records in the 6 months prior to the procedure. Medication use was recorded through personalized interviews or electronic prescription verification. CRC family history was obtained through personalized interviews. Colonoscopies were performed by expert gastroenterologists, using Olympus CF-H190 colonoscopes (Olympus Europa, Barcelona, Spain). Image-enhanced endoscopy was used to endoscopically characterize the visualized lesions. For screening colonoscopies, the necessary requirements were met, and they were performed by experienced staff. All had high rates of reaching and exploring the cecum (>90%). Histological examination was conducted by expert pathologists specifically dedicated to the study of digestive pathology. CRN was classified according to the criteria of the World Health Organization [17].

### 2.4. Definitions

Alcohol and tobacco consumption: Current drinkers were defined as adults who consume >2 drinks/day (men) or more than 1 drink/day (women). According to the definition from the National Center for Health Statistics, a current smoker is any adult who has smoked ≥100 cigarettes in their lifetime and currently smokes. A former smoker was defined as someone who has smoked ≥100 cigarettes but had quit smoking at the time of the survey. 

The normality criteria for the risk factors were established according to the “European Guidelines on Cardiovascular Disease Prevention in Clinical Practice (2016 version)” [18] and the standards of the Spanish Society of Atherosclerosis 2022 [19]. 

Average risk population of CRC: asymptomatic patients between 50 and 69 years of age with no personal or family history of adenomas or CRC.

Non-syndromic familial CRC: patients with first-degree family history of CRC in whom hereditary syndromes (polyposis and non-polyposis) were excluded through clinical criteria [14].

CRN was defined as any histologically confirmed adenocarcinoma or adenoma. Advanced CRN was defined as invasive CRC or adenoma ≥10 mm in diameter, high-grade dysplasia, significant villous component (>20%), or any combination thereof.

### 2.5. Calculation of Cardiovascular Risk

Among the derivation cohort of 1049 patients, CVR was calculated using: Framingham–Wilson [20], REGICOR (Girona Heart Registry) [21], SCORE (Systematic Coronary Risk Estimation) for low-risk countries [22], and FRESCO (Spanish risk function of coronary and other cardiovascular events) [23]; these are presented as continuous variables. 

### 2.6. Statistical Analysis

An initial descriptive analysis of all clinical variables was performed. Qualitative variables were expressed as frequencies and percentages and continuous variables as median with interquartile range (Q1–Q3). Normality was assessed using the Shapiro–Wilk test. Differences between independent groups were evaluated using the Chi-square (χ^2^) test for qualitative variables and the Mann–Whitney or Kruskal–Wallis test for continuous variables.

A multivariate logistic regression model with 10-fold cross-validation was used to create a new predictive score for advanced CRN, adjusted for all variables retrieved in the study (sex, age, first-degree family history of CRC, SBP, DBP, total cholesterol, HDL [high-density lipoprotein] cholesterol, body mass index [BMI], diabetes, smoking, and antihypertensive treatment). The logistic regression model is constructed by applying the logistic function to a linear combination of predictor variables, using the formula: P (Y = 1) = $\frac{1}{1+e^{-\left(\beta_{0}+\beta_{1}{Χ}_{1}+\beta_{2}{Χ}_{2}+\ldots +\beta_{n}{Χ}_{n}\right)}}$ where Y is the binary dependent variable, Χ_1_, Χ_2_, …, Χ_n_ are the predictor variables, and β_0_, β_1_, …, β_n_ are the coefficients to be estimated through the optimization process. The correlation coefficients are shown in Supplementary Table S1.

Once the coefficients β_0_, β_1_, …, β_n_ have been estimated, this formula is used to calculate the probability of the binary dependent variable Y being equal to 1. The estimated β coefficients in the derivation population are the ones subsequently applied in the validation population.

The discriminative ability of each score to predict advanced CRN was evaluated using the area under the receiver operating characteristic (ROC) curve (AUC) and 95% confidence intervals (CI). The DeLong test was used to test the statistical significance of the difference between the areas under two dependent ROC curves.

The significance level in this study was set at 0.05. The analyses were conducted using the R programming language v.3.5.3 (R Foundation for Statistical Computing, Vienna, Austria) [24]. 

The results obtained were validated in a different external population.

## 3. Results

### 3.1. Clinical and Demographic Characteristics of the Derivation Cohort

A total of 1049 patients were included, of whom 463 (44%) were males, with a median age of 58 years (IQR = 51.3–64.5). Overall, 36% (*n* = 379) had CRN; 151 patients (14%) had non-advanced CRN; while 228 (22%) had advanced CRN. A total of 2.8% of the patients (*n* = 30) had CRC. 

Table 1 displays the clinical and demographic characteristics of the derivation cohort. Significant differences were observed between patients with a normal colonoscopy and those with CRN: Patients with CRN were more frequently male, older, and had a higher likelihood of alcohol consumption (*p* < 0.001). They also had a higher prevalence of obesity, diabetes, and dyslipidemia (*p* < 0.05). Patients with CRN had a lower frequency of first-degree family history of CRC compared to those without colonoscopy lesions (*p* < 0.05). 

In the multivariate analysis, male sex, age, diabetes, and tobacco consumption were identified as risk factors for the development of advanced CRN (Table 2).

### 3.2. Cardiovascular Risk in the Derivation Cohort 

When calculating CVR in the derivation cohort based on colonoscopy findings, it was observed that patients with CRN had higher scores in all applied scores compared to those without lesions in endoscopy (*p* < 0.001), with the exception of the FRESCO Model A (*p* = 0.353) (Table 3). 

### 3.3. Predictive Capacity for Advanced CRN of the Cardiovascular Risk Scores

**Figure 1:**
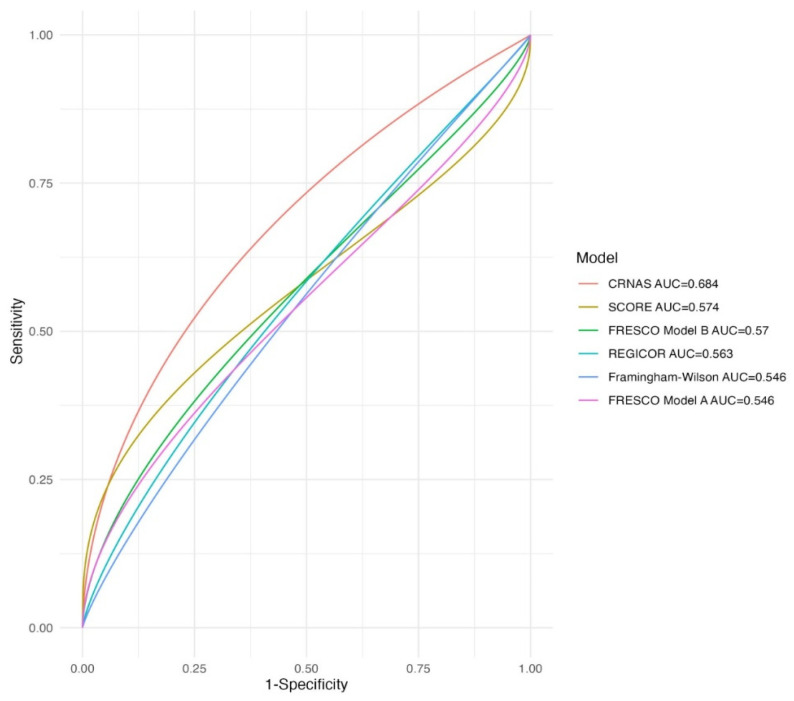
Comparison of ROC curves for different models in predicting advanced CRN (Framingham–Wilson; FRESCO Model A [Spanish risk function of coronary and other cardiovascular events]; FRESCO Model B; REGICOR [Girona Heart Registry]; SCORE [Systematic Coronary Risk Estimation]; CRNAS [ColoRectal Neoplasia Advanced Score]).

The predictive capacity to detect advanced CRN by each of the applied CVR scores is shown as ROC curves in Figure 1. The highest predictive capacity was obtained by the FRESCO Model B (AUC 0.57; 95% CI: 0.53–0.61) and SCORE (AUC 0.57; 95% CI: 0.53–0.62).

### 3.4. Predictive Capacity of the New Score 

When independently combining the presence of first-degree family history of CRC along with age, sex, and other known CVRF (BMI, SBP, DBP or use of antihypertensive treatment, total cholesterol, HDL cholesterol, diabetes, smoking) using a multivariate logistic regression model into a new score (CRNAS: ColoRectal Neoplasia Advanced Score), a significant improvement in the predictive capacity of advanced CRN (AUC 0.68; 95% CI: 0.64–0.72) was observed (Figure 1). The improvement in the detection of advanced CRN by CRNAS compared to each of the previously evaluated CVR scores was statistically significant in all cases (*p* < 0.001 for REGICOR, Framingham–Wilson; SCORE; FRESCO models A and B). 

**Figure 2:**
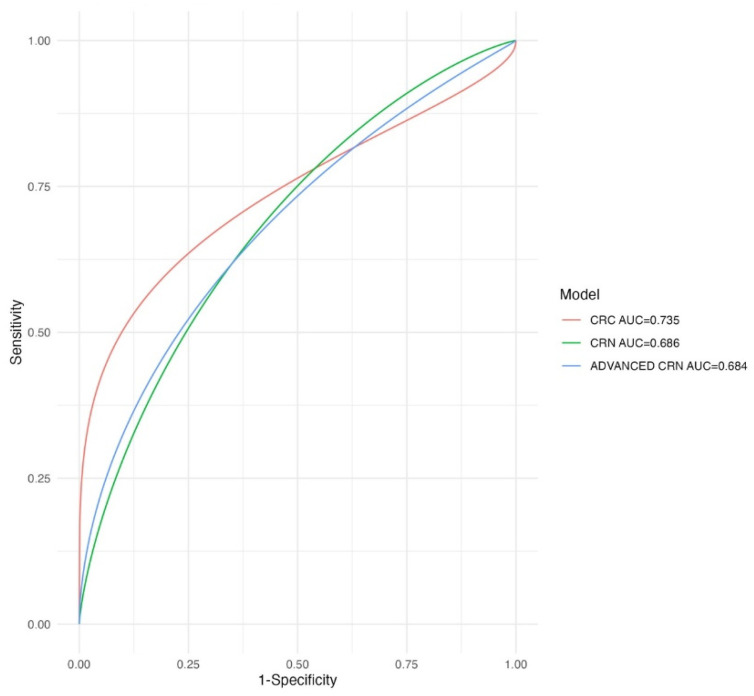
Comparison of ROC curves for CRNAS model in detecting CRC, CRN, and advanced CRN. CRC: colorectal cancer; CRN: colorectal neoplasia; CRNAS: colorectal neoplasia advanced score.

The highest predictive capacity of the CRNAS score was for the detection of CRC (AUC 0.74; 95% CI 0.63–0.84) (Figure 2).

### 3.5. Internal and External Validation of the CRNAS

The validation cohort consisted of 308 patients with a median age of 60.2 years (IQR = 50.5–67.7), with 48% males (*n* = 148). A total of 31% of the patients (*n* = 95) presented a CRN and 13% (*n* =40), an advanced CRN. The external validation cohort only showed differences between groups (normal colonoscopy, non-advanced CRN, advanced CRN) for age, HTA, and diabetes (*p* < 0.05) (data shown in Supplementary Table S2). Differences in baseline characteristics between the derivation cohort and the validation cohort are shown in Supplementary Table S3. In both populations, the prevalence of males and CRN was similar, however, the proportion of advanced CRN in the validation cohort was lower (*p* < 0.001). There was also a lower proportion of patients with alcohol, tobacco, and NSAID consumption as well as patients with first- or second-degree family history of CRC (*p* < 0.001). The median age, the prevalence of HTA, diabetes, hypertriglyceridemia, and antiplatelet usage were higher than in the derivation cohort (*p* < 0.05). CRNAS was validated both internally in the original population and externally (Table 4). The improvement in the predictive capacity of advanced CRN was maintained in this external cohort (AUC 0.67; 95% CI: 0.57–0.76).

## 4. Discussion

Both CRC and CVD are the leading causes of mortality and morbidity worldwide. Previous studies have shown a strong coexistence of CRN and CVD, probably due to shared risk factors (e.g., smoking, obesity, and metabolic syndrome) and pathophysiological mechanisms (e.g., insulin resistance, chronic inflammation, and oxidative stress) [25].

CRC is often developed from precancerous lesions so it is also one of the most preventable and curable tumors when detected early; however, the implementation of colonoscopy-based screening can be limited for several reasons, including insufficient resources, low participant compliance, or concerns about procedure-related complications. In this regard, risk prediction models could be useful in more accurately identifying high-risk individuals and implementing timely prevention measures, thereby improving their effectiveness, acceptance, and compliance.

Based on the presence of shared risk factors between CRC and CVD, CVR scores could be useful not only for predicting individual CVR but also for predicting the risk of advanced CRN. Furthermore, creating a score that combines CVRF with other risk factors linked to CRC could be highly valuable in establishing more personalized screening measures. 

In this study, we present a simple and valid score for predicting the risk of advanced CRN in a Southern European population, using age and sex, along with various CVRF, and the presence of first-degree family history of CRC. 

It should be mentioned that our risk score doesn’t fit for sigmoidoscopy screening as the primary endpoint of our model was advanced CRN located anywhere in the colorectum. Advanced CRN, not just CRC, was chosen for analysis because it has been suggested as the most appropriate target for endoscopy screening [16].

In our multivariate analysis, only age, sex, tobacco consumption, and diabetes were associated with the risk of advanced CRN. Contrary to what might be expected with the current available evidence, after adjusted analysis, family history did not behave as a risk factor for advanced CRN. It was established that first-degree relatives of CRC patients are at higher risk of developing CRN compared to the population without a family history although this has been questioned lately, at least for individuals with only one first-degree relative [26]. The risk also varies depending on the degree of kinship, age of the index case at diagnosis, number of affected family members, and sex [27].

On the other hand, in a systematic review and meta-analysis of predictive scores for advanced CRN in an average-risk population [28], age, sex, first-degree family history of CRC, BMI, and smoking were the most commonly included factors in the scores as they have been shown to be associated with the development of advanced CRN. Based on this, we decided to include them as well as risk factors associated in our multivariate analysis and others such as SBP, DBP, and cholesterol (total and HDL), widely used to calculate CVR scores. 

To date, there is limited data on the potential role of CVR scores in predicting CRN. Only Framingham and SCORE [11,12,13,29,30] have been evaluated. However, other CVR scores used and adapted to the Spanish population, such as FRESCO or REGICOR, have not been evaluated. Precisely, in our cohort, FRESCO Model B and SCORE obtained the best predictive capacity. Furthermore, there are few European studies that have evaluated CVRF along with family history of CRC to predict the risk of advanced CRN [31]. While it was not the main objective of our study, the results and application of CVR scores should be interpreted with caution because they were applied to a population not specifically collected for this purpose, including young and elderly patients, patients with known coronary artery disease, chronic kidney disease, diabetics, as well as patients with other pathologies that can modify CVR.

The predictive capacity for advanced CRN by the different CVR scores is not high (the best model obtained an AUC value of 0.57; 95% CI: 0.53–0.61). On the other hand, based on previous studies [32,33], the predictive capacity for CRC based on family history (adjusted for age or age and sex, respectively), showed an AUC ranging between 0.53 and 0.55; however, in our study, by combining the presence of family history with other CVRF, we were able to improve the predictive capacity (AUC value of 0.68; 95% CI: 0.64–0.73), and what is important, this improvement was maintained in the external validation cohort (AUC 0.67; 95% CI: 0.57–0.76). 

This AUC value was slightly better than those reported in other studies, which use predictive models based on dietary habits and lifestyle, such as the Betés score [15] (AUC 0.65 for advanced CRN; 95% CI (0.61–0.68)) or the Kaminski score [31] with an AUC of 0.62 for advanced CRN (95% CI, 0.60–0.64). Along the same line, our score also demonstrates a predictive capability better than previously developed scores based on more complex and costly genetic analyses [32,33,34]. For example, Jeon et al. [32] with a combined model of genetic, environmental, and lifestyle factors showed a discriminatory capacity of 0.63 for men and 0.62 for women. Ibáñez et al. [33] reported a similar discriminatory capacity (0.63) using genetic, environmental risk factors, and family history. Our research group reported an AUC of 0.66 with a genetic model combined with age and sex [34]. Finally, between the studies with the highest predictive capacity, Cai et al. [16] reported an AUC of 0.74 (95% CI: 0.70–0.78), using age, sex, smoking, diabetes mellitus, and the consumption of green vegetables, pickles, fried foods, and white meats; however, some these factors are prone to recall bias.

It should also be pointed out that the CRNAS score proposed in this study has been designed and validated by combining a wide range of populations, including average-risk (asymptomatic) individuals, symptomatic patients, or those with a family history of CRC who do not meet the criteria for hereditary CRC. Few studies have evaluated the risk of advanced CRN in symptomatic populations [35].

The validation cohort differed in baseline characteristics from the derivation cohort, although there were no differences in terms of sex and the difference in median age is likely not clinically relevant. 

This study has several strengths. The first of them is precisely its external validation in a population with baseline characteristics different from the derivation cohort, which would increase the reliability of the results. Another aspect is that is easy to implement in a primary care setting since all variables are easy to collect and do not need either genetic data or dietary habits. When risk scores are used in clinical or community settings, the number of predictors should be as small as possible, risk factors should be easy to obtain and there should be a balance between the simplicity of the model and the prediction accuracy. Furthermore, it shows an improvement in predictive capability to detect advanced CRN as opposed to relying solely on CVRF or even those combining genetic and dietary habits, having a moderate accuracy to predict advanced CRN in addition to being able to use some of its parameters to also calculate individual CVR.

However, our study also has limitations typical of cross-sectional studies. Regarding the recording of medication use, tobacco use, and alcohol consumption, there may be biases in data collection and memory biases that could affect the results. Finally, the observed lack of association between CRC family history and advanced CRC may be due to the fact that we did not sub-analyze the degree of kinship, age of the index case at diagnosis, number of affected family members, or sex.

In summary, this study shows that the combination of CVRF and CRC-specific risk factors improves the predictive capacity to identify patients at high risk of advanced CRN. This simple score is easy to calculate and could change the way CRC screening measures are applied, making them more individualized, avoiding the performance of colonoscopies in low-risk patients, and prioritizing it in high-risk patients. In addition, it allows calculating individual cardiovascular risk, enabling the establishment of preventive measures for this condition, which in turn, could be used as primary prevention for CRC by controlling common risk factors. Finally, our study prompts the question of whether patients with CRN have an intrinsic raised CVR compared to those without or with other non-neoplastic lesions that could explain the findings obtained in previous studies [36,37]. These findings should be confirmed in further studies and in other non-Caucasian populations before applying them to routine clinical practice. 

## Figures and Tables

**Table 1:** Clinical and demographic characteristics of this study’s derivation cohort according to colonoscopy findings.

Characteristics	Total Cohort (*n* = 1049)	No Neoplasia (*n* = 670)	Non-Advanced CRN (*n* = 151)	Advanced CRN (*n* = 228)	*p*-Value
Age (years) Median (Q1–Q3)	58 (51.3–64.5)	56 (49.2–63.7)	59.5 (54.1–63.8)	60.9 (54.7–66.4)	**<0.001**
Sex: male	463 (44%)	238 (36%)	82 (54%)	143 (63%)	**<0.001**
Tobacco					0.056
Current smoker	246 (33%)	150 (22%)	34 (23%)	62 (27%)	
Former smoker	216 (21%)	125 (19%)	34 (23%)	57 (25%)	
Alcohol consumption	368 (35%)	200 (30%)	67 (44%)	101 (44%)	**<0.001**
Obesity	243 (25%)	141 (22%)	37 (28%)	65 (30%)	**0.044**
Hypertension	260 (25%)	156 (23%)	37 (25%)	67 (29%)	0.182
Diabetes	92 (9%)	45 (7%)	13 (9%)	34 (15%)	**<0.001**
Hypercholesterolemia	567 (54%)	367 (55%)	91 (60%)	109 (48%)	**0.048**
Hypertriglyceridemia	115 (11%)	70 (11%)	11 (8%)	34 (15%)	0.058
NSAID use	484 (46%)	323 (48%)	71 (47%)	90 (39%)	0.071
Antiplatelet use	82 (8%)	49 (7%)	11 (7%)	22 (10%)	0.507
First-degree family history of CRC	375 (36%)	259 (39%)	43 (28%)	73 (32%)	**0.026**
Second-degree family history of CRC	133 (13%)	90 (13%)	17 (11%)	26 (11%)	0.621

NSAID: Nonsteroidal Anti-Inflammatory Drugs. CRN: colorectal neoplasia. CRC: colorectal cancer. Bold indicates *p*-value < 0.05; *n*: number of individuals; values expressed as *n* (%) unless otherwise indicated.

**Table 2:** Associations between individual characteristics and advanced CRN in a multivariate analysis.

Characteristics	OR	95% CI	*p*-Value
Sex: female	0.40	0.29–0.56	**<0.001**
Age	1.05	1.03–1.06	**<0.001**
Total cholesterol	1	1–1	0.7
HDL cholesterol	1	0.99–1.01	0.7
BMI	0.99	0.95–1.03	0.5
Systolic blood pressure	1	0.99–1.02	0.6
Diastolic blood pressure	1	0.99–1.03	0.3
Diabetes	1.66	1–2.71	**0.047**
Current smokers	1.55	1.07–2.24	**0.019**
Antihypertensive treatment	0.82	0.55–1.21	0.3
First-degree family history of CRC	0.98	0.7–1.37	>0.9

HDL cholesterol: high-density lipoproteins cholesterol; BMI: body mass index; CRC: colorectal cancer; OR: odds Ratio; CI: confidence interval. Bold indicates *p*-value < 0.05.

**Table 3:** Cardiovascular risk in the derivation cohort according to colonoscopy results.

Cardiovascular Risk Scores	Total Cohort (*n* = 1049)	No Neoplasia (*n* = 670)	Non-Advanced CRN (*n* = 151)	Advanced CRN (*n* = 228)	*p*-Value
Framingham–Wilson	8.5 (5.1–12.5)	7.9 (4.7–12)	9.2 (6.6–13.4)	9.3 (5.5–13.1)	**<0.001**
REGICOR	3.1 (1.9–4.7)	2.8 (1.7–4.4)	3.6 (2.4–5.2)	3.6 (2.2–5.2)	**<0.001**
SCORE	0.7 (0.2–1.5)	0.6 (0.2–1.4)	0.6 (0.3–1.3)	0.8 (0.3–1.7)	**<0.001**
FRESCO Model A	3.1 (1.6–4.7)	3.1 (1.6–4.7)	3 (1.6–4.2)	3 (1.7–5.2)	0.353
FRESCO Model B	3.2 (1.7–5.8)	3 (1.5–5.5)	3 (2.1–5.4)	3.7 (2.2–7.2)	**<0.001**

REGICOR: Girona heart registry; SCORE: systematic coronary risk estimation; FRESCO: Spanish risk function of coronary and other cardiovascular events. Values obtained as median (Q1–Q3). *n*: number of individuals. Bold indicates *p*-value < 0.05.

**Table 4:** Internal and external validation data for cardiovascular risk scores and CRNAS.

**Predictive Capacity of Advanced CRN**		
**Model**	**AUC Derivation Cohort** **(*n* = 1049)**	**AUC Validation Cohort** **(*n* = 308)**
Framingham–Wilson	0.55 (0.50–0.59)	0.51 (0.41–0.61)
REGICOR	0.56 (0.52–0.61)	0.51 (0.42–0.61)
SCORE	0.57 (0.53–0.62)	0.58 (0.48–0.67)
FRESCO Model A	0.55 (0.50–0.59)	0.55 (0.45–0.64)
FRESCO Model B	0.57 (0.53–0.61)	0.58 (0.48–0.68)
CRNAS	0.68 (0.64–0.73)	0.67 (0.57–0.76)
**Predictive Capacity of CRN**		
**Model**	**AUC Derivation Cohort** **(*n* = 1049)**	**AUC Validation Cohort** **(*n* = 308)**
Framingham–Wilson	0.56 (0.52–0.6)	0.59 (0.52–0.66)
REGICOR	0.58 (0.54–0.62)	0.59 (0.52–0.66)
SCORE	0.55 (0.52–0.59)	0.59 (0.52–0.66)
FRESCO Model A	0.52 (0.48–0.56)	0.58 (0.51–0.65)
FRESCO Model B	0.55 (0.52–0.6)	0.62 (0.55–0.69)
CRNAS	0.69 (0.65–0.72)	0.62 (0.55–0.69)
**Predictive Capacity of CRC**		
**Model**	**AUC Derivation Cohort** **(*n* = 1049)**	**AUC Validation Cohort** **(*n* = 308)**
Framingham–Wilson	0.56 (0.45–0.66)	0.55 (0.4–0.7)
REGICOR	0.57 (0.46–0.67)	0.52 (0.38–0.67)
SCORE	0.61 (0.5–0.71)	0.63 (0.5–0.78)
FRESCO Model A	0.64 (0.53–0.74)	0.55 (0.4–0.7)
FRESCO Model B	0.61 (0.51–0.72)	0.56 (0.42–0.71)
CRNAS	0.74 (0.63–0.84)	0.74 (0.59–0.88)

REGICOR: Girona heart registry; SCORE: systematic coronary risk estimation; FRESCO: Spanish risk function of coronary and other cardiovascular events; CRNAS: colorectal neoplasia advanced score; AUC: area under the ROC Curve. AUC values for CRNAS and cardiovascular risk models for internal and external validation with 95% CI. *n*: number of individuals.

## Data Availability

The original contributions presented in the study are included in the article/Supplementary Material, further inquires can be directed to the corresponding author upon reasonable request.

## Supplementary Materials

The following supporting information can be downloaded at: https://www.mdpi.com/article/10.3390/jcm13102887/s1, Table S1. The correlation coefficients for CRC, advanced CRN and CRN; Table S2: Epidemiological characteristics of the validation cohort. Table S3: Comparative table between the derivation and validation populations.
