# Supplementary material for: Predictive Score for Advanced Colorectal Neoplasia Based on Cardiovascular and Colorectal Cancer Risk Factors
Source: J Clin Med. 2024 May 14;13(10):2887. doi: 10.3390/jcm13102887 (PMC11122001; doi:10.3390/jcm13102887)
Supplement: Supplementary file 1 [file jcm-13-02887-s001.zip › jcm-2984650-supplementary.pdf]

Supplementary Table S1: The correlation coefficients for CRC, advanced CRN and CRN

COEFFICIENTS	CRC	ADVANCED CRN	CRN
Intercept	-9.555 (-15.751 : -3.728)	-4.599 (-6.911 : -2.344)	-4.682 (-6.634 : -2.774)
Sex-female	-0.769 (-1.62 : 0.038)	-0.916 (-1.252 : -0.585)	-0.959 (-1.248 : -0.673)
Age	0.079 (0.034 : 0.126)	0.045 (0.027 : 0.063)	0.039 (0.024 : 0.054)
Cholesterol	0.003 (-0.007 : 0.012)	0.001 (-0.003 : 0.005)	0.004 (0 : 0.007)
BMI	0.013 (-0.086 : 0.103)	-0.012 (-0.052 : 0.027)	0.018 (-0.015 : 0.051)
HDL-Cholesterol	0.003 (-0.025 : 0.028)	0.002 (-0.009 : 0.013)	0.001 (-0.009 : 0.01)
Systolic blood pressure	0.005 (-0.026 : 0.034)	0.003 (-0.011 : 0.017)	0.006 (-0.006 : 0.018)
Diastolic blood pressure	-0.001 (-0.049 : 0.047)	0.009 (-0.01 : 0.029)	0.005 (-0.012 : 0.022)
Diabetes	0.013 (-1.18 : 1.034)	0.504 (-0.001 : 0.997)	0.391 (-0.083 : 0.865)
Current smokers	-0.064 (-1.197 : 0.885)	0.44 (0.068 : 0.806)	0.319 (-0.008 : 0.645)
Antihypertensive treatment	0.099 (-0.793 : 0.965)	-0.193 (-0.589 : 0.195)	-0.332 (-0.679 : 0.012)
First-degree family history of CRC	-0.758 (-1.87 : 0.161)	-0.015 (-0.351 : 0.316)	-0.246 (-0.535 : 0.041)

BMI:Body Mass Index; HDL-cholesterol:High-density lipoproteins cholesterol;CRC: Colorectal cancer

Supplementary Table S2: Epidemiological characteristics of the validation cohort.

CHARACTERISTICS	TOTAL COHORT *(N=308)*	NO NEOPLASIA *(n=213)*	NON-ADVANCED CRN *(n=55)*	ADVANCED CRN *(n=40)*	*p*-value
Age (years) Median(Q1-Q3)	60.16 (50.47 - 67.65)	58 (47.96 - 66.64)	61.64 (55.71 - 68.76)	63.51 (58.69 - 69.51)	**<0.001**
Sex-Male	148 (48%)	97 (46%)	25 (45%)	26 (65%)	0.071
Tobacco					0.191
Current smoker	52 (17%)	33 (15%)	11 (20%)	8 (20%)	
Former smoker	76 (25%)	48 (23%)	13 (24%)	15 (38%)	
Alcohol consumption	52 (17%)	36 (17%)	8 (15%)	8 (20%)	0.782
Obesity	89 (29%)	59 (28%)	17 (31%)	13 (33%)	0.775
Hypertension	119 (39%)	71 (33%)	28 (51%)	20 (50%)	**0.017**
Diabetes	54 (18%)	30 (14%)	15 (27%)	9 (23%)	**0.049**
Hypercholesterolemia	139 (45%)	93 (44%)	29 (53%)	17 (43%)	0.454
Hypertriglyceridemia	94 (31%)	64 (30%)	17 (31%)	13 (33%)	0.951
NSAID use	21 (7%)	13 (6%	6 (11%)	2 (5%)	0.406
Antiplatelet use	37 (12%)	27 (13%)	7 (13%)	3 (8%)	0.685
First-degree family history of CRC	53 (17%)	36 (17%)	13 (24%)	4 (10%)	0.216
Second-degree family history of CRC	16 (5%)	12 (6%)	2 (4%)	2 (5%)	0.923

NSAID:Nonsteroidal Anti-Inflammatory Drugs. CRN: Colorectal neoplasia. CRC: Colorectal cancer

Bold indicates *p*-value <0.05.

n: number of individuals.

Values expressed as n (%) unless otherwise indicated.

Supplementary Table S3: Comparative table between the derivation and validation populations.

**CHARACTERISTICS**	**DERIVATION COHORT** *(n=1049)*	**VALIDATION COHORT** *(n=308)*	*p*-value
Age (years)			
Median (Q1-Q3)	57.96 (51.3 - 64.5)	60.16 (50.5 - 67.7)	**0.031**
Male	463 (44%)	148(48%)	0.225
Tobacco			
Current smoker	246 (23%)	52 (17%)	**0.034**
Former smoker	216 (21%)	76 (25%)	
Alcohol consumption	368 (35%)	52 (17%)	**<0.001**
Obesity	243 (25%)	89 (29%)	0.151
HTA	260 (25%)	119(39%)	**<0.001**
Diabetes	92 (9%)	54 (18%)	**<0.001**
Hypercholesterolemia	567 (54%)	139 (45%)	0.365
Hypertriglyceridemia	115 (11%)	94 (31%)	**<0.001**
NSAID use	484 (46%)	21 (7%)	**<0.001**
Antiplatelet use	82(8%)	37(12%)	**0.022**
First-degree family history of CRC	375 (36%)	53 (17%)	**<0.001**
Second-degree family history of CRC	133 (13%)	16 (5%)	**<0.001**
Colorectal neoplasia	379 (36%)	95 (31%)	0.087
Advanced colorectal neoplasia	228(22%)	40(13%)	**<0.001**

HTA: Hypertension. NSAID:NonSteroidal Anti-Inflamatory Drugs. CRN: Colorectal neoplasia. CRC: Colorectal cancer

Bold indicates *p*-value <0.05.

n: number of individuals.

Values expressed as n (%) unless otherwise indicated.
